# Diabetes distress in Barbadian adults with type 2 diabetes

**DOI:** 10.3934/publichealth.2022032

**Published:** 2022-05-23

**Authors:** Allison DaSantos, Carlisle Goddard, Dalip Ragoobirsingh

**Affiliations:** 1 University of the West Indies, Faculty of Medical Sciences, Cave Hill Campus Cave Hill Bridgetown, Barbados; 2 University of the West Indies Faculty of Medical Sciences, Bridgetown, Barbados; 3 Professor-Medical Biochemistry and Diabetology, Director-UWI Diabetes Education Programme, University of the West Indies Faculty of Medical Sciences Teaching & Research Complex (Level 2) Mona, Kingston 7, Jamaica

**Keywords:** diabetes distress, diabetes distress scale, type 2 diabetes, Barbados, adults

## Abstract

**Purpose:**

Psychological disorders such as diabetes distress may negatively influence how patients effectively manage their illness. Therefore, discernment of such influential psychosocial components could be pertinent in promoting competent diabetes management. The vital psychosocial aspect of diabetes distress in diabetes management within the Caribbean context remains unexplored. The purpose of this study therefore was to investigate the prevalence and distribution of diabetes distress (DD), and explore its relationship to socio-demographic and clinical characteristics in Barbadian adults with type 2 diabetes(T2DM).

**Methods:**

A cross-sectional survey was conducted with adults (n = 509) ages 20–80 years with T2DM. The survey comprised, a profile section, and a standardized questionnaire—the Diabetes Distress Scale (DDS). In addition, biological (A1C and blood pressure measurements were also collected.

**Results:**

The sample comprised 30.8% males and 69.2% females. Moderate to high DD identified in 17% of the patients (9.4% moderate distress, 7.2% high distress). Moderate distress was more frequent in unmarried persons; younger persons had high DD. There was no difference in rates of moderate to high DD in women (16.7%) compared to men (16.2%) and, as age and years lived with diabetes increased, diabetes distress decreased.

**Conclusions:**

The results emphasized the need for considerations that must be given to Barbadian diabetes patients' mental well-being. In recognition of the role DD plays in the patients' experiences, screening should be incorporated into clinical care.

## 1. Introduction

In Barbados, non-communicable diseases are estimated to account for 83% of all deaths; with a proportional mortality from diabetes of 9% [1]. In addition, related complications of diabetes increase the burden of the disease in the country [2]. Further compounding the problem is the presence of at least one co-morbid condition such as; cardiovascular disease, cerebrovascular disease, renal disease, or hypertension. Moreover, possibly adding to the overall impact of morbidity and mortality is that patients may also suffer from coexisting psychological issues such as diabetes distress (DD).

Existing evidence, points specifically to DD as a major factor associated with treatment outcomes and mental well-being of diabetic patients. Notwithstanding the existence of research literature on this aspect of diabetes treatment, there is in the Caribbean, a paucity of research in this area. In the Caribbean, research on diabetes has primarily focused on epidemiological statistics such as prevalence and incidence of diabetes, its risks factors and its complications [3]–[5]. Yet, there is no literature that supports that there is a problem with psychological disorders such as DD in persons with diabetes in Barbados. However, that the outcomes for diabetes is less than optimal [2], may be indicative that these variables may be associated with outcomes, as studies in other countries have demonstrated the probable relationships.

The research into diabetes related emotional distress and quality of life has highlighted the definite association between emotional and mental well-being and diabetes. Representative of this is existing evidence that points specifically to the phenomena diabetes distress; an emotional state in which persons living with diabetes feel guilt, stress and or denial as a result of having to manage their illness.

Cross sectional studies have determined that, diabetes related distress is associated with concurrent diabetes self-management [6]–[9], glycemic control [6],[9],[10], and treatment adherence [11]. Further, studies indicate that DD is linked to physical activity and A1C [12]. In other studies of patients with type 2 diabetes, the positive association between negative insulin appraisal and DD was considerably high [13].

Diabetes is considered a psychologically and behaviorally challenging disease demanding consistently high levels of adherence in order to achieve lower morbidity. Patients are therefore required to adapt their lifestyle practices (including diet, exercise), and this may inevitably affect their disposition. Thus, psychosocial factors are relevant to its management.

In consideration of this, the issue of, “diabetes distress”, having been defined and identified as a major negative influencing factor in diabetes management is of significant import. This vital aspect in diabetes management within the Caribbean context was previously left unexplored. Thus, the major goal of this study was to investigate, for the first time (according to available data), in a Caribbean setting, the challenges posed by DD in good diabetes control. This may be relevant, as studies have demonstrated the potentiality of interventions to enhance therapeutic outcomes for diabetes, but these are generally subsequent to the contextual comprehension of the factors involved in the phenomena.

The purpose of this study therefore, was to investigate the prevalence and distribution of diabetes distress in people with known T2DM in Barbados.

## 2. Materials and methods

This study was undertaken, employing a cross-sectional survey which sought to describe the frequency of the particular attribute (DD) within the defined population (persons with T2DM) over an eight-month period. The aim of the survey was to gather information about the present and past occurrences and to investigate the associations between the variables.

### 2.1. Participants

The study was conducted in Barbados and the target population was individuals in the 20 to 80 years age group who had been diagnosed with type 2 diabetes and who were registered at public polyclinics in the country. Public polyclinics are decentralized healthcare facilities offering a wide range of healthcare services to the general population on a daily basis. There are nine publicly subsidized polyclinics providing primary healthcare in Barbados, and these are located strategically around the island within easy access to their catchment areas. The required sample of patients from the polyclinics was drawn using proportional stratified sampling technique. In the current study, the determined strata were the polyclinics attended by patients, as individuals are registered to only one polyclinic. The sampling fraction was determined to be 0.5 as the constant proportion ratio for each population subset. The desired sample size was based upon the combination of available resources and the desired precision.

#### 2.1.1. Sample size calculations

Sample size calculations were conducted using the Epi Info 7 software. To estimate the proportion of type 2 diabetes mellitus patients with diabetes distress, with a 95% confidence interval of ± 5%, 384 subjects were required. An additional number of persons were sampled to make up for any participant dropout.

Permission was sought and acquired from the Institutional Review Board of the University of the West Indies at Cave Hill and The Ministry of Health of Barbados, for the investigator to conduct research within the public polyclinics throughout the island. The inclusion criteria were: persons diagnosed with T2DMs [14] between the ages of 20 and 80 years, an absence of severe diabetes complications or functional deficits (dialysis, blindness), and no diagnosis of psychosis or dementia.

### 2.2. Recruitment procedure

Persons attending the polyclinics are usually seen by a nurse(s) prior to their consultation with the physician. These nurses are therefore able to identify from their charts the patients who are diagnosed with T2DM. The nurse(s) then informed the investigator, who then systematically randomized those patients to approach and requested their participation in the study. The investigator approached potential participants in the waiting area and discretely requested their participation. All participants were assured that their participation was completely discretionary and that if they choose, they may withdraw at any time. All who agreed to participate signed a consent form.

### 2.3. Materials

For the collection of quantitative data, a descriptive survey design in the form of a questionnaire battery (paper-pencil form) was utilized to obtain participants' self-reports. This direct method of data collection is a quick, easy, simple, inexpensive and feasible method [15],[16] to use within the given constraints of available time and resources.

The questionnaire comprised of two parts Part One: encompassed questions on socio-demographic characteristics such as: sex, age, marital status; as well as questions on clinical characteristics such as: presence of co-morbid disease and disease related complications, and duration of illness. Part Two: consisted of: The Diabetes Distress Scale (DDS) [17], which sought to screen for and measure the level of diabetes distress.

The presence and degree of diabetes distress was determined by participants' scores on the (DDS). The scale yields a total diabetes distress score for each participant, by summing each participant's response to the 17 items and dividing by the number of items on the scale. Three categories of (DD) are then define according to the scores; “little or no DD” (DDS < 2.0), “moderate DD” (DDS = 2.0–2.9), and “high DD” (DDS ≥ 3.0) [18]. The DDS also yields four sub-scales, each addressing a different kind of distress [19]. These are calculated as follows; Emotional Burden: mean of 5 items (1, 4, 7, 10, and 14); Physician Distress: mean of 4 items (2, 5, 11, and 15); Regimen Distress: mean of 5 items (6, 8, 3, 12, and 16) and Inter Personal Distress: mean of 3 items (9, 13, and 17).

Additionally, biological (A1C and blood pressure) measures were collected prior to the administration of the survey.

## 3. Results

The population of this study consisted of 509 persons diagnosed with T2DM and registered at the public polyclinics on the island. Although 572 persons where approached, 555 individuals fit the criteria for eligibility, of which, 511 persons agreed to participate, with 509 providing viable data (critical elements of data were missing from 2 participants). Quantitative data collected were then analyzed using SPSS (Statistical Packages for Social Sciences) version 24.0 for Windows software. Each statistical test was two-sided and a p-value of ≤ 0.05 was deemed statistically significant.

The overall sample in the dataset for analysis consisted of, 30.8% males (n = 157) and 69.2% females (n = 352) with an overall mean age of 63.54 (SD ± 11.73). See Table 1 for the frequency of the demographic and clinical variables of participants

**Table 1–1.** Frequency of demographic and clinical variables.

Clinical variable	N	Percent
Any Amputations	509	100.0
Blood Test for Diabetes in three years	509	100.0
Take Tablets	421	82.7
Take Insulin	143	28.1
Kidney Disease	28	5.5
Stroke	33	6.5
Eyes Affected	107	21.0
Heart Failure	48	9.4
High Cholesterol	295	58.0
Hypertensive	405	79.6
Lived Outside Barbados	124	24.4
Diabetes Managed outside Caribbean	46	9.0
Know A1C Levels	34	6.7

**Table 1–2.** Frequency of demographic and clinical variables.

Demographic variable		N	Percent
Gender	Male	157	30.8
	Female	352	69.2
	Total	509	100.0
Marital Status	Married	162	31.8
	Legally Separated	24	4.7
	Never been Married	205	40.3
	Divorced	49	9.6
	Widowed	62	12.2
	Other	7	1.4
	Total	509	100.0
Highest level of Education completed	No formal Schooling	4	0.8
	Less than Primary School	34	6.7
	Primary School Completed	193	37.9
	Secondary School Completed	182	35.8
	Technical/Secretarial after Primary School	8	1.6
	Technical/Secretarial after Secondary School	48	9.4
	Teacher Education	3	0.6
	College/University completed	36	7.1
	Post Graduate Degree	1	0.2
	Total	509	100.0
Work Activity	Worked	169	33.2
	With Job not working	8	1.6
	Looked for work	9	1.8
	Home Duties	34	6.7
	Student	2	0.4
	Retired	240	47.2
	Incapacitated	47	9.2
	Total	509	100.0

**Table 1–3.** Frequency of demographic and clinical variables.

Descriptive Statistics	N	Mean	Std. Deviation
Age in Years	509	63.54	11.173
Number of Years Diagnosed	508	13.507579	10.5466984
How many medications Use	509	1.76	0.928
Systolic Blood Pressure (mm/Hg)	509	137.73	20.128
Diastolic Blood Pressure (mm/Hg)	509	81.56	11.027
Glycated Haemoglobin level	509	8.270	2.1355

For the 509 participants the majority of persons 83.4% had little distress (n = 425), while 9.4% had moderate distress (n = 47) and 7.2% had high distress (n = 37). The DDS mean score for this sample as a whole was 1.54 (SD ± 0.76). The mean scores of the sample as a whole for the subscales were: Emotional burden (M = 1.59, SD ± 0.92); Physician Distress (M = 1.26, SD ± 0.66); Regimen Distress (M = 1.76, SD ± 0.94) and Inter Personal Distress (M = 1.44, SD ± 1.01).

For the male participants (n = 154), 83.8% had little distress (n = 129), 9.1% had moderate distress (n = 14) and 7.1% had high distress (n = 11). For female participants (n = 346), 83.2% had little distress (n = 288), 9.5% had moderate distress (n = 33) and 7.2% had high distress (n = 25).

Levels of high DD is most prevalent in participants who reported never been married (n = 19). These patients also had the highest prevalence for moderate levels of DD (n = 22). Within the education levels the group with the greatest prevalence in high DD were those participants who reported Primary School completion as their highest level of education (n = 14).

Spearman's Rho analysis, revealed:

1. A positive significant correlation between years lived with diabetes diagnosis and age, rs = 0.31 n = 508, p < 0.01. This suggests that these two variables are linearly related, that more years lived with diabetes diagnosis was associated with older age.

2. A medium negative significant correlation between Age and DD, rs = −0.31 n = 509, p < 0.01, Suggesting that as age increases, DD scores decreases, or younger persons experience higher levels of DD. There was a large (strong) significant negative correlation between number of years diagnosed and DD scores, rs = −0.91, n = 509, p < 0.05, suggesting that with less years of diagnosis was associated with high levels of DD.

3. A small negative correlation between the number of years with diabetes and the DDS sub-scale Regimen Related Distress, rs = 0.12, n = 508, p < 0.01, with low levels of regimen related distress associated with more years living with diabetes.

4. A small positive correlation between A1C and the DDS, rs = 0.25, n = 509, p < 0.01, with high diabetes distress associated with high A1C.

A standard multiple regression was used to assess the ability of age in years and number of years diagnosed to predict levels of DD. The model which included age and number of years diagnose explains 9% of the variance in DD with statistical significance (p < 0.001). Of the two variables Age made a statistically significant contribution (β = −0.30, p < 0.001). Years of diagnosis however did not make a statistically significant contribution (β = 0.01, p > 0.05). Indicating that as age increases by one standard deviation (SD = 11.17) the DD Scores would decrease by 0.30 standard deviation units. Multiple Regression with two predictors R^2^ = 0.09, F (2, 505) = 24.75, p < 0.001.

A Mann-Whitney U test revealed; (a) no significant difference in DD scores for males (Md = 1.24, n = 157) and females (Md = 1.24, n = 352), U = 27359, Z = −0.18, p = 0.86, r = 0.01. (b) no significant difference in DD scores for married (Md = 1.18, n = 162) and unmarried (Md = 1.29, n = 347), U = 25407, Z = −1.76, p = 0.08, r = 0.08.

To determine if there was a difference in the DD scores for participants in the under 45, 45–65 and over 65 age groups. A one-way between groups analysis of variance was conducted to explore the impact of age on levels of distress as was measured by the Diabetes Distress Scale (DDS). Participants were divided into three groups according to their age (Group 1: under 45years; Group 2: 45 to 65years and Group 3: 65years and above. There was a statistically significant difference at the p < 0.05 level in DDS scores for the three age groups: F (2, 262) = 18.83, p < 0.05. The actual difference in mean scores between the groups was medium according to Cohen's classification [20]. The effect size calculated using eta squared, was 0.10. Post-hoc comparisons using the Tukey HSD test indicated that the mean score for Group 1 (M = 2.40, SD ± 1.04) was significantly different from Group 2 (M = 1.63, SD ± 0.82) and Group 3 (M = 1.4, SD ± 0.57), and Group 2 differed significantly from Group 3. (Additionally, the assumption of homogeneity of variances was tested and demonstrated a violation via the Levene F test, F (2) = 20.58, p < 0.05. (Brown-Forsythe Robust Test of Equality of Means was reported).

A chi-square test for independence indicated no significant association between Gender and levels of Diabetes Distress, χ^2^ (2, n = 509) = 0.03, p = 0.99, Cramer's V = 0.01 (small) association between the variables. See Table 2.

**Table 2.** Gender and levels of diabetes distress.

Level of Distress
Gender	Little	Moderate	High
Male	83.8%	9.1%	7.1%
Female	83.2%	9.5%	7.2%

A chi-square test indicated a significant association, between age groups and DD χ^2^ (4, n = 500) = 32.13, p < 0.05. Specifically, for persons in the under 45 age group reported high distress greater than would be expected. Residual = 7.2, Z = 5.4 (greater than critical value 1.96).

## 4. Discussion

The present study documents evidence of the presence of DD among adults with T2DM within this Barbadian sample. In this study, moderate to high DD was identified in 17% of the patients with type 2 diabetes (9.4% measured as experiencing moderate distress while 7.2% had high distress). Fisher and colleagues [21] stated that even though patients with diabetes emotional distress levels have not reached the depression rates that necessitates medical attention, patients suffering from elevated emotional distress levels was because they were in constant worry due to their chronic illness condition. The results of the current study while similar to the one conducted by Aljuaid and colleagues [22], which reported a prevalence of 25% in moderate to high DD in T2DM patients; differed from most other previous studies in that the prevalence of distress were lower. For example, studies conducted in Malaysia [23], China [11], and Sudan [24] showed a prevalence of DD of 49.2%, 64% and 87.6% respectively. Comparison between these studies and the current study may be disproportionate due to the unique cultural, social, and economic context this study may have relative to the previous studies. Furthermore, there were differences in the sample and demographics of these studies in proportion to the current study as well as their existing health and welfare systems and policies.

In the current study, there was no difference in the rates of moderate to high DD in women (16.7%) when compared to men (16.2%). This is in contrast to the results from the study conducted by Ali and colleagues [25], which found that DD was higher in women (23.8%) than in men (12.8%). Furthermore, a systematic review highlighted that DD was significantly greater in those samples that had a majority of women when compared to samples that had a majority of men. The authors posited that this increase in the existence of emotional distress in women with T2DM could be ascribed to the difference in social conventions relating to gender, where men seem to a smaller extent, to ask for help or admit to distress because of the desire to give the impression that they are capable or, due to the fear of being emasculated by appearing weak. Another argument presented, was that men may try to effectuate the idea of the masculine identity, in particular, problem solving, thereby leading them to procure advice in order to overcome their condition [26]. In the current study, however, these stereotypes appear to differ or may even be absent, further investigation may be necessary to determine what, if any, cultural gender norms influence DD in men and women within this Caribbean context.

In this study, it was found that as age and years lived with diabetes increased, diabetes distress decreased suggesting that DD was higher in younger patients, as well as those persons who lived less time with diabetes diagnosis. This may be due to their strength and degree of awareness having lived with the illness for less time, they are still adjusting. The Transtheoretical Model acknowledges that change in behavior is a process that occurs over a period through a series of stages, rather than just as an event. Moreover, these changes may occur linearly through the stages, but often progress may be non-linear, and most individuals may revert and/or repeat to the earlier stages. Further, the longer persons have lived with illness the more aware they are about the disease. For example, in an application of this theory to persons living longer with the illness, these stages would include the preparation stage—in which persons intend to take action in the immediate future, after having taken some significant action in the recent past; the action stage—where persons have made specific obvious changes in their lifestyle and maintenance stage—where persons not only make obvious changes in their life style but also endeavor to prevent relapses. It may be possible that they have “passed through” varying stages, having had a longer time to adjust, thus affecting their emotional and subsequent selfcare levels. Younger patients and those living shorter period with illness diagnosis in contrast may be in the earlier stages of Pre-contemplation—no intention to take any action in the foreseeable future and Contemplation-getting ready to make changes in near future. This may be reflective of their degree of self-efficacy or confidence that they may have regarding their ability to maintain the desired behavior change.

The results also found that high and moderate distress was most prevalent in participants who were never married, 9.3% and 10.7% respectively. This may be due to the perceived lack of social support that could assist them in managing their daily life, even more, help with managing their illness. Meta-analysis of the impact of social support, found that there was a reduction in anxiety, and a development of feelings of security when individuals experienced positive social communication with family members and friends. Moreover, high social support protects individuals from illnesses [27].

There was a difference in the DD scores between the age groups with younger patients demonstrating higher DD. This result was also reported by Wardian and Sun, in their study of the factors related to DD [28]. Persons within this age category as members of a work force, must deal with managing the stresses of work, family and finances along with managing their illness, making daily life even more stressful; whereas older patients may not have to face these same challenges on a daily basis and so may be better able to concentrate and thus cope with their diabetes.

This study however is not without limitations. Interpretations of the results must be considered within the context of the design; due to the cross-sectional design of the study, causal inferences cannot be examined, nor the impermanence of the effects. Future longitudinal work may be necessary to elucidate the probability of directionality and causality in the associations. The generalizability of the results is limited by first, the health-care setting of the study, (public health care clinics), and second the specific disease (type 2 diabetes).

## 5. Conclusions

The results suggest that the presence of this emotional disorder may be insufficiently acknowledged in Barbadian patients with type 2 diabetes mellitus, as 17% were identified as having diabetes distress. The results of a review on affective disorders in diabetic patients, demonstrated that in order to achieve optimal results, treatment should include special consideration of psychological and psychiatric knowledge, as the pertinent behavior of the patient will determine how effective therapy is; because the occurrence of emotional disorders affects social functioning and negatively affects the course of the illness [29]. Some collaboration therefore, between physicians and mental health professionals may be efficacious for the all-round health care of persons living with diabetes.

The presence of DD among Barbadian patients with T2DM emphasizes the need for considerations that must be given to the individual's emotional and mental well-being, and, in recognizing the roles this factor plays in the patients' experiences, actions may be taken to address this sometimes seemingly subtle influencer of outcomes. Thus, any endeavours aimed at reducing barriers to effective diabetes management could benefit from the inclusion of screening for DD.

All persons diagnosed with diabetes should be assessed for distress, with considerations given to severity. Additionally, DD should be assessed routinely utilizing a validated screening instrument (DDS). The incorporation of this into standard medical care would be an acknowledgement of the potential impact of life stressors on diabetes management and the normalizing of DD as a part of living with diabetes.

Priority should be given to addressing diabetes distress in the early stages of clinical care as this is likely to be more effective due to the ‘malleability’ of diabetes distress, particularly when at low to moderate levels. Such an approach would support the better quality of life, self-care and health outcomes associated with well-timed detection and the management of emotional distress.

For cases of moderate distress, more focused interventions are recommended; for example; family interventions or structural problem solving. And for, higher levels of distress, more aggressive interventions, inclusive of psychotherapy and/or pharmacotherapy, while still considering the context of diabetes. This approach would however necessitate a coordinated multidisciplinary combination of expertise (physiological and psychological) to provide comprehensive care.
